# Expression Analysis, Functional Marker Development and Verification of *AgFNSI* in Celery

**DOI:** 10.1038/s41598-019-57054-x

**Published:** 2020-01-17

**Authors:** Jun Yan, Lizhong He, Shuang Xu, Yanhui Wan, Hong Wang, Ying Wang, Li Yu, Weimin Zhu

**Affiliations:** Horticulture Research Institute, Shanghai Academy of Agricultural Sciences; Key Laboratory of Protected Horticulture Technology, Shanghai, 201403 China

**Keywords:** Plant breeding, Plant molecular biology

## Abstract

Apigenin is one of the primary flavonoids in celery, which has a high medicinal value. Flavone synthase I (FNSI) is the last step enzyme in apigenin biosynthesis. In this study, the 1492 bp promoter sequence before *AgFNSI* initiation codon (ATG) of celery was obtained, which included methyl jasmonate (MeJA) responsive elements, light responsive elements, anaerobic induction elements and five MYB binding sites. *AgFNSI* was sensitive to temperature, UV-B, water deficit and MeJA. Comparative analysis of *AgFNSI* genome and promoter sequences among celery accessions with different apigenin content showed that there were four allelic variations in *AgFNSI*, and four accessions with high apigenin content belonged to AgFNSIa, and five accessions with low apigenin content belonged to AgFNSIc. Three pairs of dominant complementary markers were designed based on the single-nucleotile polymorphisms (SNPs) of the AgFNSIa and AgFNSIc genomes and promoter sequences. Three pairs of functional markers were validated by 112 celery accessions. The results showed that AFPA1/AFPB1 detected significant differences in apigenin content between different genotypes. Therefore, marker AFPA1/AFPB1 is associated with apigenin content in celery and could be used for the genetic improvement of apigenin content in celery.

## Introduction

Improvements in living standards have fostered increased awareness of dietary healthy. Vegetable varieties with significant functional components have become important targets for breeding and agricultural production. Apigenin is one of the primary flavonoids in celery, which has a high medicinal value. It possesses important physiological functions such as anticancer^1^, neuroprotection^2^, regulation of blood lipids^3^. In addition, apigenin has been shown to improve the stress resistance of plants^4^. Apigenin is thus closely associated with the stress resistance and health qualities of celery.

Apigenin is an intermediate product of flavonoid metabolism, which is synthesized by flavone synthase I (FNSI) using naringenin as substrates^5^. FNSI belongs to 2-oxoglutarate-dependent dioxygenase (2-ODD), which is an important enzyme involved in a variety of oxidative reactions. 2-ODD is involved in the synthesis of ethylene, gibberellin and flavonoids, including gibberellin 20 oxidase (GA20ox), flavanone 3-hydroxylase (FHT), anthocyanidin synthase (NS) and FNSI^6^. Current research shows that the 2-ODD family has only 19–75% nucleic acid and amino acid sequences conservation; for example, the homology of the nucleic acid and amino acid sequences of the gibberellin synthase gene in different species is only 50– 60%^7^. Gebhardt *et al*.^8,9^. found that there was an 80% sequence similarity between *FNSI* and *FHT* in Umbelliferae plants, and that *FNSI* was generated by the mutation of seven amino acids during the evolution of *FHT*. Chen *et al*.^10^ analyzed the *FNSI* sequence differences of three different celery varieties and found five mutation sites. It was reported that apigenin content varies remarkably among different varieties of celery, and our preliminary studies showed that the expression of *FNSI* was positively correlated with the accumulation of apigenin^11^. Environmental stress and hormone treatments can result in the changes in plant secondary metabolites^12^. At present, the expression pattern of *FNSI* in celery under environmental stress and plant hormone treatments and its relationship with apigenin accumulation have not been reported.

Functional markers are recent development based on the phenotypic differences caused by the polymorphic sequences of target genes and are able to determine the existence of target alleles in a variety of genetic backgrounds^13^. Therefore, exploring the characteristics of the *FNSI* gene and developing corresponding functional markers should improve the accuracy of phenotypic prediction and the efficiency of molecular -assisted breeding, which is of great significance for improving celery quality. In this study, real-time fluorescence quantitative PCR (qRT-PCR) was used to analyze the expression patterns of *AgFNSI* in celery under different environmental stress and plant hormone treatments. The promoter sequence of *AgFNSI* in celery was obtained by homologous cloning, and the *cis*-acting elements of the promoter were analyzed by bioinformatics. Specific primers were designed according to the difference in genome and promoter sequences between four high-apigenin and five low-apigenin content accessions to explore the functional markers closely linked to apigenin content. Then, 112 celery accessionswere used to validate the effectiveness of the functional markers. Finally, functional markers were developed to detect the allelic variation associated with apigenin accumulation in celery.

## Results

### Expression pattern of *AgFNSI* under different stress and hormone conditions

**Figure 1:**
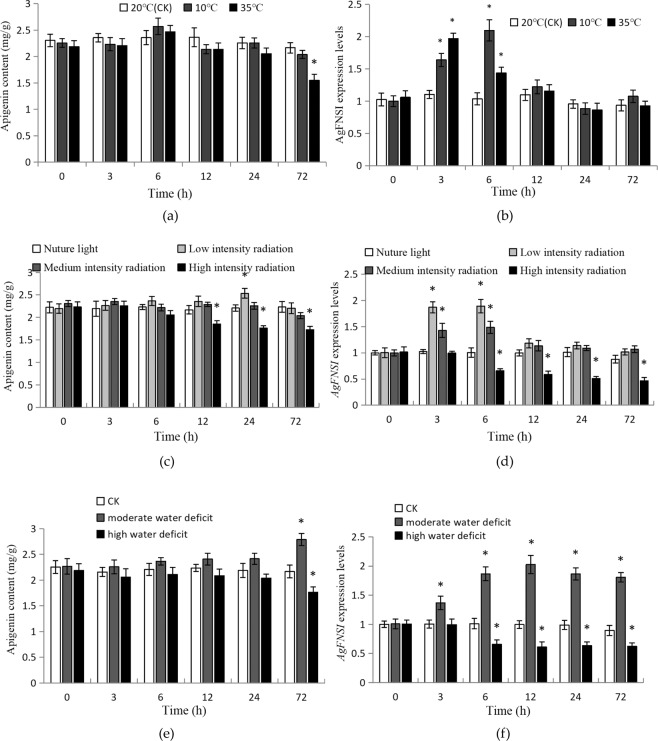
The apigenin contents and the expression levels of AgFNSI in celery leaves after treated with different temperatures (**a,b**), UV-B radiation intensity (**c,d**) and different osmotic potentials (**e,f**) for 3 h, 6 h, 12 h, 24 h and 72 h.The data points are mean of three biological replicates ± SD. The asterisk show significant differences.

**Figure 2:**
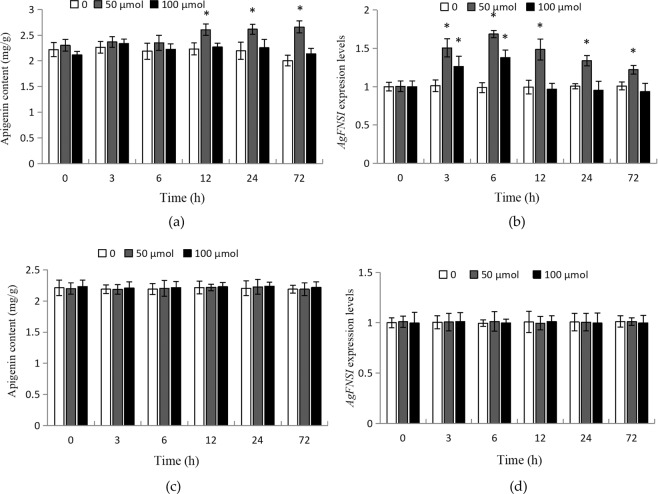
The apigenin contents and the expression levels of *AgFNSI* in celery leaves after treated with different MeJA contents (**a,b**) and SA (**c,d**) contents for 3 h, 6 h, 12 h, 24 h and 72 h.The data points are mean of three biological replicates ± SD. The asterisk show significant differences.

The expression patterns of *AgFNSI* in response to different stress and hormone treatments were detected by qRT-PCR, and the changes in apigenin content were also measured. The results showed that both high- and low-temperature treatments could affect the expression of *AgFNSI*. After 3 and 6 h of treatment, the expression levels of *AgFNSI* increased significantly, following which they declined (Fig. 1b). There was no significant change in the apigenin content under the low-temperature treatment, and the apigenin content decreased significantly after 72 h of high-temperature treatment (Fig. 1a). The expression levels of *AgFNSI* significantly increased after low- and medium-intensity UV-B treatment for 3 h and 6 h, but were significantly reduced after 6 h of high-intensity UV-B treatment (Fig. 1d). The apigenin content increased significantly after 24 h of low-intensity UV-B treatment, but decreased after 12 h of high-intensity UV-B treatment (Fig. 1c). After 3, 6, and 12 h moderate water deficit, the expression levels of *AgFNSI* increased significantly, decreasing slightly thereafter. High water deficit significantly decreased the expression levels of *AgFNSI* (Fig. 1f). Apigenin content increased significantly after 72 h moderate water deficit, but decreased significantly after 72 h High water deficit (Fig. 1e). *AgFNSI* was insensitive to SA (Fig. 2d), but was up-regulated by MeJA (Fig. 2b). The expression levels of *AgFNSI* increased significantly after MeJA treatment, especially the 50 μmol MeJA treatment. Additionally, the content of apigenin increased significantly 50 μmol MeJA treatment for 12 h (Fig. 2a). The results showed that *AgFNSI* was sensitive to temperature, UV-B, water deficit and MeJA, and long-term treatment could affect the apigenin content.

### Cloning and analysis of the *AgFNSI* promoter sequence

To further explore the regulatory mechanism of *AgFNSI*, a 1492-bp promoter sequence upstream of the *AgFNSI* initiation codon(ATG) was obtained, and was analyzed by PlantCARE. The results indicated that these sequences contained typical elements of eukaryotic promoter A-box (2), CAAT-box (23) and TATA-box (56), which are able to bind to initiation transcription factors. In these sequences, the Box 4, GATA-motif, GT1-motif, T-box, and TCT-motif elements are related to light reaction; ARE and GC-motif are *cis*-acting elements related to anaerobic or hypoxic induction; and the CGTCA-motif and TGACG-motif are *cis*-acting elements related to MeJA. Metal-binding sites (MBS) are binding sites of MYB transcription factors related to drought induction. In addition, MYB, MYB-like sequence, and Myb are binding sites of MYB transcription factors related to unknown functions, and some unknown functional elements were also detected (see Supplementary Table S1).

### Sequence analysis and marker development of *AgFNSI*

The genome and promoter sequences of *AgFNSI* in the 13 celery accessions were comparative analyzed by DNAMAN5.0 software. Four allele mutation sequences were obtained, namely as AgFNSIa, AgFNSIb, AgFNSIc and AgFNSId (see Supplementary Fig. S1). In these four allele mutation sequences, there were 29 SNP locis and eight insertion deletion (In/Del) locis in the promoter region, and 25 SNP locis and three In/Del locis in the genome region (see Supplementary Table S2). Among these, there were 10 A/T types, 12 A/G types, 24 C/T types, three A/C types,three T/G types and one G/C type. In five high apigenin accessions (HAA), the *AgFNSI* sequences of four HAA belonged to AgFNSIa, while one belonged to AgFNSIb. In eight low apigenin accessions (LAA), the *AgFNSI* sequences of one LAA belonged to AgFNSIb, five LAAs belonged to AgFNSIc, and two LAAs belonged to AgFNSId. There were three amino acid mutations among four sequences. The protein sequences of AgFNSIa and AgFNSIb were identical. Compared with AgFNSIc, two SNPs caused the mutation of the 4^th^ and 144^th^ amino acids, which changed from threonine (AgFNSIa, AgFNSIb) to serine (AgFNSc) and tyrosine (AgFNSIa, AgFNSIb) to cysteine (AgFNSc), respectively. Compared with AgFNSId, in addition to the above two amino acids, the 38^th^ amino acid changed from isoleucine (AgFNSIa, AgFNSIb) to valine (AgFNSId).

**Figure 3:**
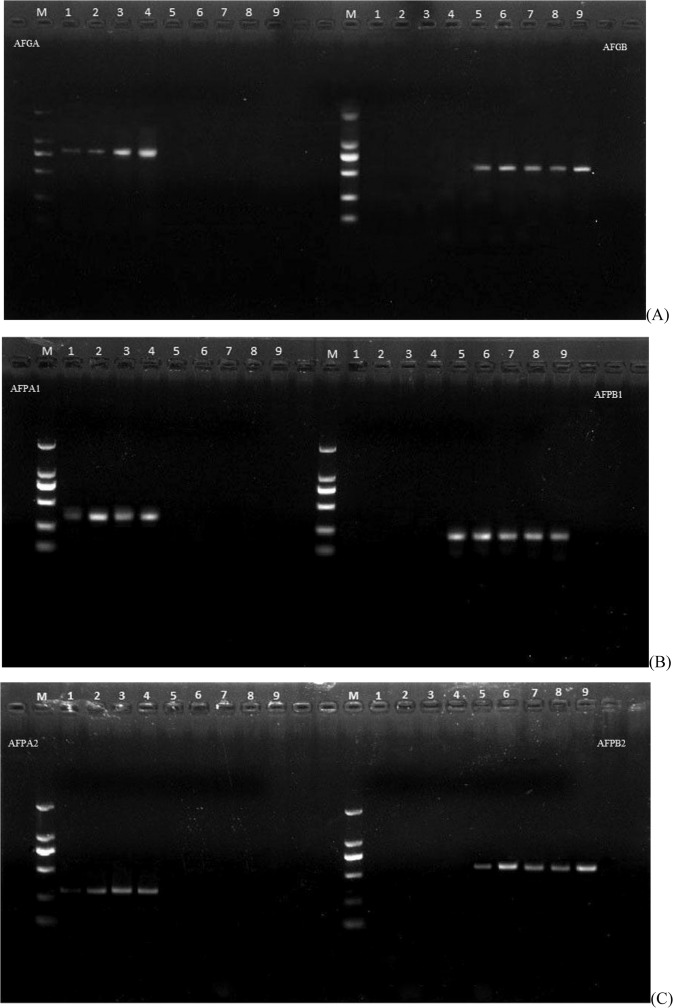
Polymorphic test of PCR fragments amplified by AFGA/AFGB (**A**), AFPA1/AFPB1(**B**) and AFPA2/AFPB2 (**C**) markers in four high-apigenin accessions and five low-apigenin accessions. M: DNA ladder DL2000(2000bp, 1000 bp, 750 bp, 500 bp, 250 bp, 100 bp); 1: Zhangqiu Bao Qin(3228 mg/kg); 2: Huangxin Qin (3126 mg/kg); 3: Xingjiang Small Mao Qin (2967 mg/kg); 4: Yuxiang No. 1(2940 mg/kg); 5: Ventura (1134 mg/kg); 6: California King (1157 mg/kg); 7: Prince (1138 mg/kg); 8: Rome P9 (1109 mg/kg); 9: Holy Emperor (1134 mg/kg). The numerical value in parentheses indicates apigenin content.

A pair of dominant complementary marker (AFGA/AFGB) was designed based on three SNP loci at 420–441 bp of the *AgFNSI* genome sequences between four AgFNSIa in the HAAs and five AgFNSIc in the LAAs. AFGA could amplify 770 bp fragments in four HAAs (Fig. 3A), but not in five LAAs, while AFGB could amplify 598 bp fragments in LAAs, but not in HAAs (Fig. 3A). A pair of dominant complementary marker (AFPA1/AFPB1) was designed based on the three SNP loci located at 105–121 bp in the promoter region of the nine accessions mentioned above. AFPA1 could amplify 353 bp fragments in HAAs (Fig. 3B), but not in LAAs, while AFPB1 can amplify 230 bp fragments in LAAs, but not in HAAs (Fig. 3B). A pair of dominant complementary marker (AFPA2/AFPB2) was designed based on the three SNP loci located at 794–802 bp in the promoter region of the nine accessions mentioned above. AFPA2 could amplify 323 bp fragments in HAAs (Fig. 3C), but not in LAAs, while AFPB2 could amplify 559 bp fragments in LAAs, but not in HAAs (Fig. 3C).

### Verification of functional markers

The apigenin content of the 112 celery varieties from the Fengxian and Songjiang experimental bases in Shanghai was determined in 2016 and 2017. In 2016, the content range of apigenin from the Fengxian base and Songjiang base was 1056–3128 mg/kg and 1089–3068 mg/kg, respectively. In 2017, the content range of apigenin from Fengxian base and Songjiang base was 1156–3328 mg/kg and 1208–3359 mg/kg, respectively. The average data of the two bases in 2016 were generally lower than that in 2017.

**Figure 4:**
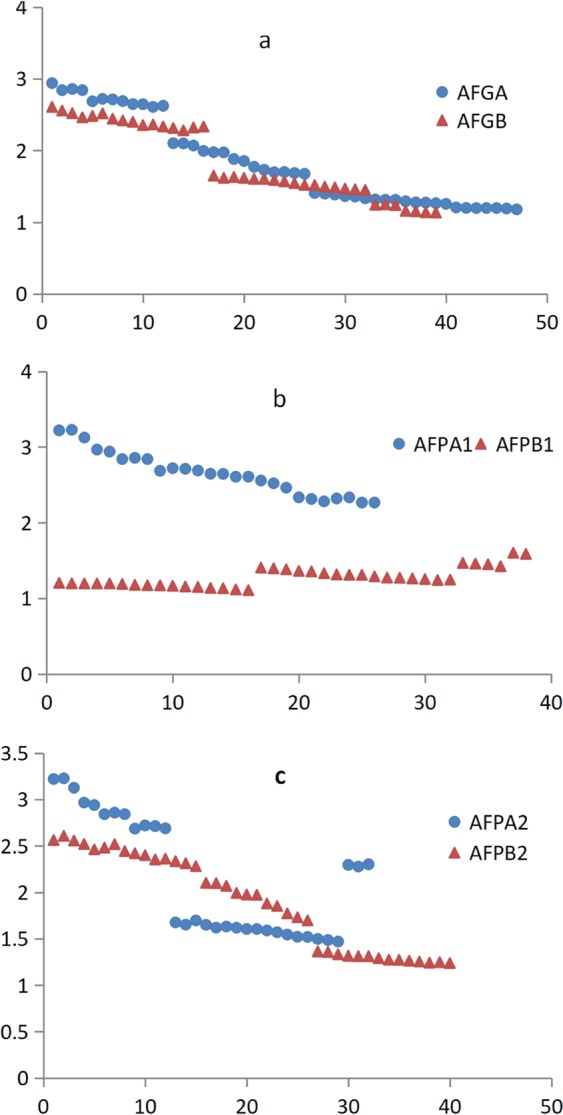
The distribution of apigenin content in the detection results of 112 celery accessions labeled with three markers.

Combined with the apigenin content results, the effectiveness of the three candidate markers was validated by PCR amplification in 112 celery accessions. A pair of markers AFGA/AFGB designed with SNPs in an intron of the genome was tested in 112 accessions, and the results showed that 47 accessions amplified 770 bp fragments, while 39 accessions amplified 598 bp fragments (Fig. 4a), and the other accessions do not amplified any fragment. The average apigenin content of these 47 AgFNSIa genotypes was 1828 mg/kg, while in the 39 AgFNSIc genotypes, the corresponding values was 1843 mg/kg (Table 1). There was no significant difference in apigenin content among the two genotypes. Two pairs of markers (AFPA1/AFPB1 and AFPA2/AFPB2) were designed using SNPs in the promoter region. The results of AFPA2/AFPB2 detection in the 112 accessions showed that 32 accessions amplified 323 bp fragments, while 40 accessions amplified 559 bp fragments and the other accessions do not amplified any fragment. The average apigenin content of these 32 AgFNSIa genotypes at the two bases was 2145 mg/kg, and in the 40 AgFNSIc genotypes, the corresponding values were 1897 mg/kg (Table 1). The difference between these two genotypes reached significant levels. The distribution of apigenin content show AFPA2/AFPB2 could not identify high- or low- apigenin accessions (Fig. 4c). The results of AFPA1/AFPB1 detection in the 112 accessions showed that 26 accessions amplified 353 bp fragments, while 38 accessions amplified 230 bp fragments and the other accessions do not amplified any fragment. The average apigenin content of these 26 AgFNSIa genotypes was 2654 mg/kg, while for the 38 AgFNSIc genotypes the values were 1282 mg/kg (Table 1). The difference between these two genotypes reached significant levels, and the apigenin content in each of the 26 AgFNSIa genotypes was significantly higher than each of the 38 AgFNSIc genotypes (Fig. 4b). Therefore, the marker AFPA1/AFPB1 is correlated with apigenin content and the accuracy rate of this marker is 57.14%. The marker AFPA1/AFPB1 thus can be effectively applied to molecular marker assisted selection breeding to improve the accuracy of apigenin content phenotype prediction.

**Table 1:** Statistical analysis of detection results of 112 celery accessions labeled with three markers.

Marker	Allele	No.	Mean of apigenin (mg/kg)	Range of variation
AFGA/AFGB	AgFNSIa	47	1828a	1178–2940
	AgFNSIc	39	1843a	1134–2609
AFPA1/AFPB1	AgFNSIa	26	2654a	2267–3228
	AgFNSIc	38	1282b	1109–1604
AFPA2/AFPB2	AgFNSIa	32	2145a	1469–3228
	AgFNSIc	40	1897b	1239–2609

Mean behind the different letters represent the average difference between two alleles is significant(P < 0.05).

## Discussion

Internal (often developmental) and external (environmental) signals serve as important regulators of enzyme gene expression via cis-regulatory motifs, thus controlling the production and specific accumulation of secondary metabolites^14^. Through promoter prediction, it was found that the promoters of *AgFNSI* include not only core elements of the CAAT-box and TATA-box, but also MeJA-responsive elements (CGTCA-motif, TGACG-motif), light–responsive elements (Box 4, GATA-motif, GT1-motif, T-box and TCT-motif), and anaerobic induction–related elements (ARE, GC-motif). In addition, there is one drought– induced MYB binding site, two MYB binding sites with unknown functions, and two MYC binding sites with unknown functions in the promoter region of *AgFNSI*. It was reported that the regulation of phenylpropanoid synthesis occurs as a result of the coordinated transcriptional regulation of structural genes by several DNA-binding factors including MYB, bHLH, bZIP, WRKY, MADS box, and WD40 TFs^15^. In purple-fleshed sweet potato, the promoter sequence of the anthocyanin biosynthesis related gene *IbUF3GT* contained MYB and MYC binding sites, and light, SA and gibberellic acid (GA) responsive elements^16^. The rice flavonoid pathway genes, *OsDfr* and *OsAn*s, are induced by dehydration, high salt, and ABA, and contain stress responsive promoter elements that interact with transcriptional activator, *OsC1-MYB*^17^. Transient expression assays showed that *FeMYBF1* activated the promoter of buckwheat genes and was related to anthocyanin and proanthocyanidin synthesis^18^. Therefore, the expression of *AgFNSI* might be activated by light, anaerobic stress, drought, and MeJA through interacting with MYB or MYC.

When plants suffer environment threats such as water deficit, salinity, temperature, and exposure to UV radiation, the accumulation of secondary metabolites, including volatile oils, flavonoids, alkaloids, glycosides, tannins, and resins, can ensure the survival, persistence, and competitiveness of the plants^19^. In this study, 60-day-old celery seedlings were treated with temperature, UV-B, water deficit, and MeJA and SA. The results showed that *AgFNSI* was sensitive to temperature, UV-B, water deficit, and MeJA. Short-term low temperature, high temperature and low- and medium- UV-B treatment significantly increased the expression of *AgFNSI*, and moderate water deficit could continuously increase the expression of *AgFNSI*. It was reported that UV-B radiation significantly increased the expression levels of favonoid biosynthesis related genes and the contents of flavonoids in chili pepper (*Capsicum annuum* L.)^20^ and *Chrysanthemum morifolium*^21^. An appropriate degree of water deficit can promote the accumulation of secondary metabolic products by stimulating the expression and activities of the key enzymes involved in secondary metabolism, such as baicalin biosynthesis in *Scutellaria baicalensis*^22^, flavonoid biosynthesis in wheat^23^, and terpenoid biosynthesis in sage^24^. In sorghum, high-temperature conditioning reduced the content of luteolinidin and apigeninidin^25^.

*AgFNSI* is not sensitive to salicylic acid, but is up-regulated by MeJA. MeJA promotes anthocyanin accumulation by inducing the expression of positive transcription factors and upregulating anthocyanin structural genes in apple^26^. In tea, MeJA could greatly activate secondary metabolism pathways, especially volatiles^27^. The content of apigenin increased significantly after 24 h and 72 h of stress treatment and MeJA treatment, which indicated that the expression of *AgFNSI* was correlated with the accumulation of apigenin under environmental change. Based on the analysis of the regulatory elements in the promoter region of *AgFNSI*, it is inferred that *cis*-acting elements in the promoter region play an important role in the regulation of expression in *AgFNSI*.

In this study, four allele mutation sequences (AgFNSIa, AgFNSIb, AgFNSIc, and AgFNSId) were detected in two genotypes exhibiting significant differences in apigenin content. AgFNSIb existed in both genotypes. The most direct effect of allelic mutations is the change in protein sequence, which may affect the activity of its enzyme, thus affecting its phenotype^28^. However, some allelic mutations only occur in the intron region, and do not affect protein expression^29^. The allelic mutation in this study resulted in a total of three amino acid mutation sites. Among them, the protein sequences of AgFNSIa and AgFNSIb were identical. Compared with AgFNSIc, two SNPs caused the mutation of the 4^th^ and 144^th^ amino acids, which changed from threonine (AgFNSIa, AgFNSIb) to serine (AgFNSc) and tyrosine (AgFNSIa, AgFNSIb) to cysteine (AgFNSc), respectively. These four amino acids are all polar and neutral amino acids, thus possessing the same physical and chemical properties. Compared with AgFNSId, in addition to the above two amino acids, the 38^th^ amino acids are changed from isoleucine (AgFNSIa, AgFNSIb) to valine (AgFNSId). These two amino acids are non-polar, hydrophobic amino acids, thus possessing the same physical and chemical properties. Therefore, there is little possibility that the differences in these three amino acids will lead to great differences in enzyme activity, and the mutation of the AgFNSI protein is thus not likely to cause a great change in the phenotype.

Functional markers are molecular markers used to distinguish and predict alleles and relative traits, and are designed based on the internal sequences of genes. The SNP T/G in the 5′-regulatory region of rice *qSH1* was highly correlated with grain dropping^30^. The *Dwarf8*^31^ gene and *tb1*^32^ gene in maize, which control plant height and blooming; the *TaGW2*^33^ gene and *Ppd-D1*^34^ gene in wheat, which are related to grain width and photoperiod; and *fw2.2*^35^ gene in tomato which controls fruit size were successfully used to design functional markers. Both this study and our previous studies showed that the expression levels of *AgFNSI* were correlated with the accumulation of apigenin. Therefore, according to the principles of primer design, three pairs of dominant complementary markers (AFGA/AFGB, AFPA1/AFPB1, and AFPA2/AFPB2) were designed based on the SNP sites in the promoter and intron region sequences of AgFNSIa and AgFNSIc. The 112 celery accessions were selected and amplified with three pairs of markers. The results showed that 26 AgFNSIa genotypes and 38 AgFNSIc genotypes were obtained by AFPA1/AFPB1 amplification, respectively. The apigenin contents of 26 AgFNSIa genotypes and 38 AgFNSIc genotypes differed significantly. Although the accuracy rate of this marker to verify apigenin content in celery accessions is 57.14%, we suggested that AFPA1/AFPB1 could be used as a functional marker for the identification of allelic mutations related to apigenin content in celery. It was speculated that apigenin content is a quantitative trait and is controlled by several genes. Our previous study^36^ showed that an *AgMYB1* is positively related with apigenin content and the expression of *AgFNSI*. In the future, we need to develop more new molecular markers to promote the accuracy rate of identification.

The 26 AgFNSIa genotypes included 24 local celery varieties in China and two accessions in Turkey, and the 38 AgFNSIc genotypes mainly originate from the United States, Japan, Netherlands, and France. Celery originated in Mediterranean coastal marshes and was originally characterized by a unique, pungent odor, resulting in its use in medicines and perfumes in ancient times. At the end of the 17^th^ century and the beginning of the 18^th^ century, the cultivation and selection of celery were improved, which resulted in the petioles gradually becoming widened and the odor weakening. The ornamental cultivation of celery began in China in the Han Dynasty, following which it gradually expanded in scope as a food. Through continuous selective breeding, Chinese celery with a strong odor and slender petiole was formed, which is distinct from the short and thick petiole celery cultivated abroad^37^. FNSI was derived from the FHT mutation. These belong to the ODD gene family, which is lowly conserved. Therefore, it is presumed that *AgFNSI* was subject to selective pressure during the process of celery breeding, and the allele mutation distinguished by the AFPA1/AFPB1 marker is related to the accumulation of apigenin.

## Methods

### Plant material and experimental treatment

The material used for the cloning of the *AgFNSI* promoter, stress treatments, and hormone treatments were Chinese celery variety “Huangxinqin”, which was planted in an artificial climate chamber, and the plant growth conditions were described in our previous report^11^. Sixty-day-old seedlings were treated with temperature, UV-B, water deficit, and hormones. The temperature treatment conditions were 10 °C, 20 °C(CK) and 35 °C; UV-B treatment conditions were under natural light, low intensity UV-B radiation (0.2 W/m^2^), medium intensity UV-B radiation (0.5 W/m^2^) and high intensity UV-B radiation (0.8 W/m^2^) for 8 h per day; the water deficits were induced bypolyethylene glycol 6000 (PEG-6000) solution having osmotic potentials 0(CK), −0.3 MPa (moderate water deficit) and −0.6 MPa (high water deficit); and the hormone treatment conditions were 0, 50, 100 μmol jasmonic acid (MeJA) and 0, 50, 100 μmol salicylic acid (SA),which were sprayed on the seedlings every 12 h. All treatments were repeated three times. Leaves in the second leaf position were sampled after 3, 6, 12, 24, and 72 h treatment for RNA extraction and apigenin determination.

Thirteen celery accessions (five with high apigenin content and eight with low apigenin content) were used for functional marker development, and 112 celery accessions that were obtained from the U.S. National Plant Germplasm System and from the collect of Shanghai academy of Agricultural Sciences(see Supplementary Table S3) were used for functional marker verification. The above materials were planted in the greenhouses of the Zhuanghang Experimental Base and Songjiang Experimental Base of the Facilities Horticulture Research Institute of Shanghai Academy of Agricultural Sciences in 2016 and 2017. The plant spacing was 20 cm and the row spacing was 25 cm. Stage 4 leaves in the harvesting period plant were sampled for DNA extraction and apigenin content determination. The determination method of apigenin was based on Yan *et al*.^11^. SPSS 19(IBM Corp., Armonk, NY, USA) was used for statistical analysis.

### Cloning of the *AgFNSI* promoter sequence

According to the *AgFNSI* DNA sequence (NCBI ID: MH939187), the 1500-bp region upstream of *AgFNSI* was obtained. Primer A (F: ACGATTGAGATTGTTTTGACGA, R: TGCTAATACCACAAACATACCCT) was designed by Primer Premier 5.0 software (Premier Biosoft International, Palo Alto, CA). The genomic DNA sequence of celery was used as a template, and the 20-μL PCR amplification system was included 2 μL template, 10 μL 2 × GC buffer, 0.4 μL10 mmol/L dNTPs, 4 μL 2 μmol/L specific primer, 0.2 μL rTaq DNA polymerase, and ddH_2_O. The PCR amplification procedure was 95 °C 3 min, followed by 35 cycles of 95 °C 30 s, 58 °C 30 s, 72 °C 45 s, and a final extension of 72 °C 3 min, then storage at 4 °C. The PCR products were detected by electrophoresis on 1% agarose gel. The promoter sequence of the gene was obtained by cloning and sequencing. The obtained promoter sequence was submitted to PlantCARE (http://bioinformatics.psb.ugent.be/webtools/plantcare/html/)for *cis*-acting element prediction.

### qRT-PCR analysis

Total RNA was extracted from the above samples and transcribed into cDNA. The qRT-PCR primers (F:GGGGAAGGTGGATGGAGAAA; R: TTCATTGTCTGTTCGGCCAG) were designed by Primer 3, and the celery glyceraldehyde-3-phosphate dehydrogenase (GAPDH) RNA gene was used as an internal reference. qRT-PCR was performed on an ABI system using Fast Start Universal SYBR Green Master Mix (Roche, Basel, Switzerland). The PCR cycling parameters and calculation method were the same as those described in our previous report^11^. Three biological repeats were set up, and a significance test was carried out using a double tailed equal variance *t*-test (*P* < 0.05).

### SNP and haplotype analysis, and functional marker development

The *AgFNSI* full-length sequence and promoter sequence of the 13 celery accessions were amplified using primers (F: ATGGCTCCATCAACTATAAC, R: CTGCCCTGGCAATCTCCG) and primers A, respectively. The PCR products were cloned and sequenced. DNA sequences were analyzed using the Sequman, Editseq, and MegaAlign software packages of DNA Star Software system, which included splicing, sorting, SNP, and haplotype analysis. To distinguish haplotype with different apigenin contents, primers were designed according to the SNPs of these haplotype sequences (Table 2). The size of the amplified products using these primers should be observable, such that the results of the electrophoresis can be easily and quickly analyzed. The developed functional markers were validated using the 112 celery accessions.

**Table 2:** The primers based on functional markers developed with different allelic variations.

Allele	Marker	Primer sequence (5′-3′)	Size of PCR fragment (bp)	PCR annealing temperature (°C)
*AgFNSIa,c*	AFGA	F:TGATGCTATACTTTGTCGGTGT	770	59
		R:GCAAAATTGGTAGAGAGAGAGGG		
*AgFNSIa,c*	AFGB	F:CGATGTAATACTTTGTCGGTGA	598	60
		R:CCGTATGCCTTCTGACACCT		
*AgFNSIa,c*	AFPA1	F:ACGATTGAGATTGTTTTGACGA	353	57
		R:GGGGCGAAATTTAGAATAACGA		
*AgFNSIa,c*	AFPB1	F:ACCGATTGAGATTATTTGGACGA	230	60
		R:ACCCTTTGCCTGCCTCAATA		
*AgFNSIa,c*	AFPA2	F: AGAATCTTAACCAATCCCGTACA	323	58
		R:AGTGGAGAGAAGTAGTGGATGT		
*AgFNSIa,c*	AFPB2	F: AGAATCTAAACCAACTCCGTACA	559	59
		R:TCCTCTTGTTCAACTGCACAG		

## Supplementary information

Figure S1.

Table S1.

Table S2.

Table S3.

## Data Availability

All data generated or analysed during this study are included in this published article (and its Supplementary Information Files).
