# Supplementary material for: Expression Analysis, Functional Marker Development and Verification of AgFNSI in Celery
Source: Sci Rep. 2020 Jan 17;10:531. doi: 10.1038/s41598-019-57054-x (PMC6969063; doi:10.1038/s41598-019-57054-x)
Supplement: Supplementary file 1 — Figure S1. [file 41598_2019_57054_MOESM1_ESM.pdf]

Expression Analysis, Functional Marker Development and Verification of *AgFNSI* in Celery

Jun Yan, Li Yu, Lizhong He, Shuang Xu, Yanhui Wan, Hong Wang, Ying Wang, Weimin Zhu

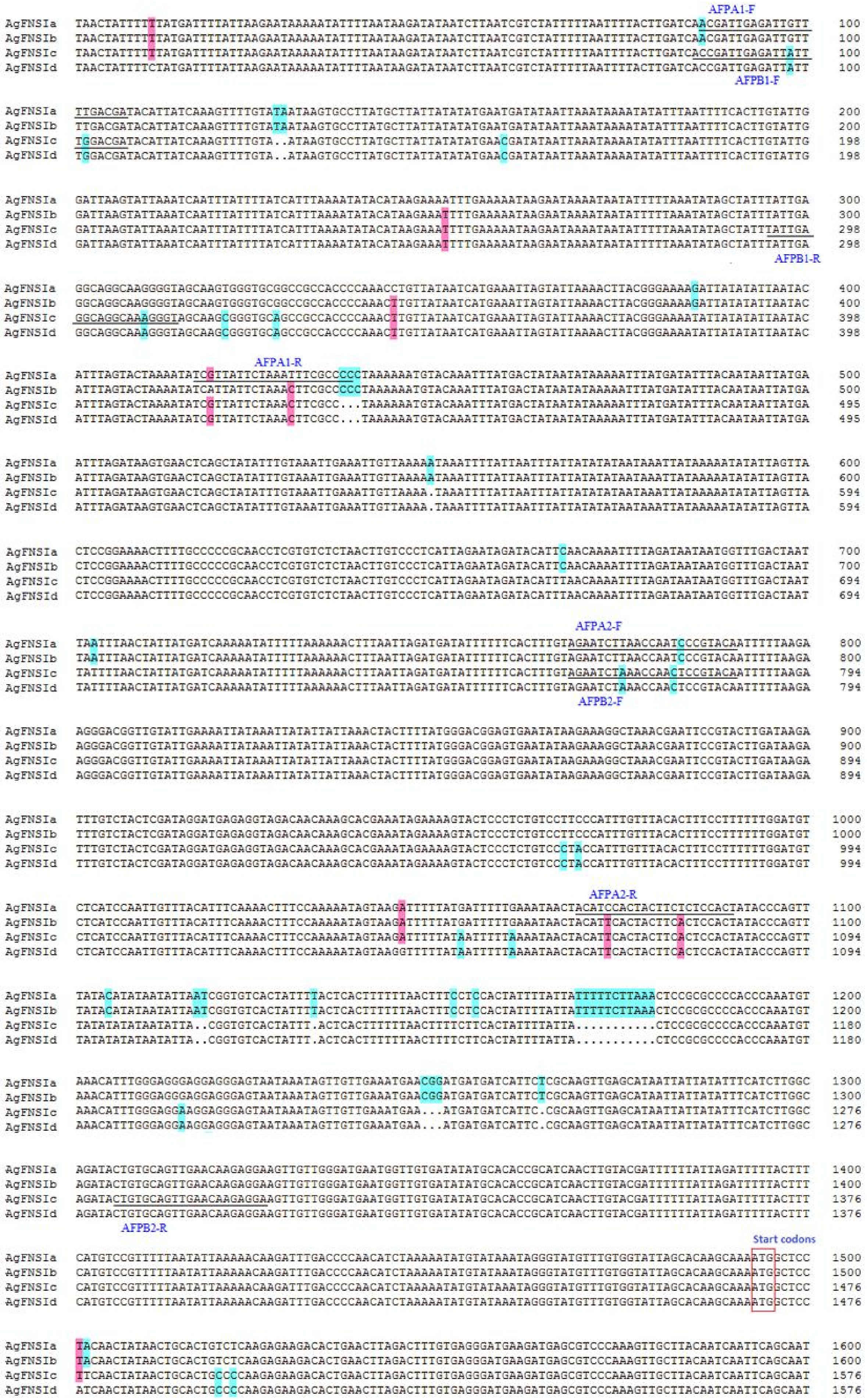

AgFNSIa
AgFNSIb
AgFNSIc
AgFNSId
AFPA1-F
AFPB1-F
AFPB1-R
AFPA1-R
AFPA2-F
AFPB2-F
AFPA2-R
AFPB2-R
Start codons

```
AgFNSIa   GAAATTCCCATCATTTCTTTAGCTGGTTTGGATGACGATTCTAATGGCAGGAGAGCTGAGATATGTCGTAAAATAGTTGAGGCTTTCGAAGAATGGGGAA   1700
AgFNSIb   GAAATTCCCATCATTTCTTTAGCTGGTTTGGATGACGATTCTAATGGCAGGAGAGCTGAGATATGTCGTAAAATAGTTGAGGCTTTCGAAGAATGGGGAA   1700
AgFNSIc   GAAATTCCCATCATTTCTTTAGCTGGTTTGGATGACGATTCTAATGGCAGGAGAGCTGAGATATGTCGTAAAATAGTTGAGGCTTTCGAAGAATGGGGAA   1676
AgFNSId   GAAGTTCCCATCATTTCTTTAGCTGGTTTGGATGACGATTCTAATGGCAGGAGAGCTGAGATATGTCGTAAAATAGTTGAGGCTTTCGAAGAATGGGGAA   1676

AgFNSIa   TTTTCCAAGTTGTTGATCACGGTATTGATAGCGGTTTGATTTCTGAGATGAGTCGTCTTTCTCGTGAATTCTTCGCTTTGCCTGCTGAGGAAAAACTTGT   1800
AgFNSIb   TTTTCCAAGTTGTTGATCACGGTATTGATAGCGGTTTGATTTCTGAGATGAGTCGTCTTTCTCGTGAATTCTTCGCTTTGCCTGCTGAGGAAAAACTTGT   1800
AgFNSIc   TTTTCCAAGTTGTTGATCACGGTATTGATAGCGGTTTGATTTCTGAGATGAGTCGTCTTTCTCGTGAATTCTTCGCCTTGCCTGCTGAGGAAAAACTTGT   1776
AgFNSId   TTTTCCAAGTTGTTGATCACGGTATTGATAGCGGTTTGATTTCTGAGATGAGTCGTCTTTCTCGTGAATTCTTCGCCTTGCCTGCTGAGGAAAAACTTGT   1776

AgFNSIa   GTATGATACCACTGGTGGAAAGAAAGGCGGCTTTACTATCTCCACTCATCTTCAGGTACGTACATTTTACCAATTTTTCCACAATTAACATCTTCAGCAA   1900
AgFNSIb   GTATGATACCACTGGTGGAAAGAAAGGCGGCTTTACTATCTCCACTCATCTTCAGGTACGTACATTTTACCAATTTTTCCACAGTTTACATCTTTAGCAA   1900
AgFNSIc   GTATGATACCACTGGTGGAAAGAAAGGCGGCTTTACTATCTCCACTCATCTTCAGGTACGTACATTTTACCAATTTTTCCACAATTAACATCTTCAGCAA   1876
AgFNSId   GTATGATACCACTGGTGGGAAGAAAGGCGGCTTTACTATCTCCACTCATCTTCAGGTACGTACATTTTACCAATTTTTCCACAATTAACATCTTCAGCAA   1876

                        AFGA-F
AgFNSIa   TAATGCATGCATGATGCTATACTTTGTCGGTGTAACATATTATTATCGATGTAACACGGAAAAATTTTATGTTTGGTTTGGTTAAACTTCAAGGGAGACA   2000
AgFNSIb   TAATGCATGCATGATGCTATACTTTGTCGGTGTAACATATTATTATCGATGTAACACGGAAAAATTTTATGTTTGGTTTGGTTAAACTTCAAGGGAGACA   2000
AgFNSIc   TAATGCATGCACGATGTAATACTTTGTCGGTGAAACATATTATTATCGATGTAACACGGAAAAATTTTATGTTCGGTTTGGTTAAACTTCAAGGGAGACA   1976
AgFNSId   TAATGCATGCACGATGTAATACTTTGTCGGTGAAACATATTATTATCGATGTAACACGGAAAAATTTTATGTTCGGTTTGGTTAAACTTCAAGGGAGACA   1976
                       AFGB-F

AgFNSIa   AACAAATTTATGGTGAAGGTATACATTCCAAGGGTAATAATTTAGATCAATTTATTTGCTTCTAATGGTTCTCGATCACTAGCAAATTTTATTATGTTGG   2100
AgFNSIb   AACAAATTTATGGTGAA..TATACATTCCAAGGGTAATAATTTAGATCAATTTATTTGCTTCTAATGGTTCTCGATCACTAGCAAATTTTATTATGTTGG   2098
AgFNSIc   AACAAATTTATGGTGAA..TATACATTCCAAGGGTAATAATTTA..TCAATTTATTTGCTTCTAATGGTTCTCGATCACTAGCAAATTTTATTATGTTGG   2072
AgFNSId   AACAAATTTATGGTGAA..TATACATTCCAAGGGTAATAATTTA..TCAATTTATTTGCTTCTAATGGTTCTCGATCACTAGCAAATTTTATTATGTTGG   2072

AgFNSIa   ACAATT..CACTAATTACCCACTCAGAGTGAATCAGACAGTCCTGTTCTTGTAATTAGCTGCTTGTTAGCGATTTTAATTATGTAAGATGATAAATATAT   2198
AgFNSIb   ACAATT..CACTAATTACCCACTCAGAGTGAATCAGACAGTCATGTTCTTGTAATTAGCTGCTTGTTAGCGATTTTAATTATGTAAGATGAGAAATATAT   2196
AgFNSIc   ACAATTAACACTAATTACCCACTCAGAGTGAATCAGACAGTCATGTTCTTGTAATTAGCTGCTTGTTAGTGATTTTAATTATGTAAGATGAGAAATATAT   2172
AgFNSId   ACAATTAACACTAATTACCCACTCAGAGTGAATCAGACAGTCATGTTCTTGTAATTAGCTGCTTGTTAGTGATTTTAATTATGTAAGATGAGAAATATAT   2172

AgFNSIa   GGAGTTATATACAGGGAGATGATGTTCGGGATTGGCGTGAGTTTGTTACTTACTTTTCGTATCCAACCAGTGCTCGGGACTACTCAAGATGGCCTAAAAA   2298
AgFNSIb   GGAGTTATATACAGGGAGATGATGTTCGGGATTGGCGTGAGTTTGTTACTTACTTTTCGTATCCAACCAGTGCTCGGGACTACTCAAGATGGCCTAAAAA   2296
AgFNSIc   GGAGTTATATACAGGGAGATGATGTTCGGGATTGGCGTGAGTTTGTTACTTACTTTTCCTATCCAACTAGTGCTCGGGACTGCTCAAGATGGCCTAAAAA   2272
AgFNSId   GGAGTTATATACAGGGAGATGATGTTCGGGATTGGCGTGAGTTTGTTACTTACTTTTCCTATCCAACTAGTGCTCGGGACTGCTCAAGATGGCCTAAAAA   2272

AgFNSIa   GCCCGAGGGGTGGAGATCAACCACGGAGGTTTATAGTGAGAAGTTAATGGTGCTAGGTGCCAAGTTACTGGAGGTGTTATCCGAGGCAATGGGGCTTGAG   2398
AgFNSIb   GCCCGAGGGGTGGAGATCAACCACGGAGGTTTATAGTGAGAAGTTAATGGTGCTAGGTGCCAAGTTACTGGAGGTGTTATCCGAGGCAATGGGGCTTGAG   2396
AgFNSIc   GCCCGAGGGGTGGAGATCAACCACGGAGGTTTATAGTGAGAAGTTAATGGTGCTAGGTGCCAAGTTACTGGAGGTGTTATCCGAGGCAATGGGGCTTGAG   2372
AgFNSId   GCCCGAGGGGTGGAGATCAACCACGGAGGTTTATAGTGAGAAGTTAATGGTGCTAGGTGCCAAGTTACTGGAGGTGTTATCCGAGGCAATGGGGCTTGAG   2372

AgFNSIa   AAAGAGGCTCTTACAAAGGCTTGTGTGGAAATGGAACAGAAAGTGTTAATTAATTACTATCCCACATGCCCCGAACCCGACCTGACGCTAGGTGTCAGAA   2498
AgFNSIb   AAAGAGGCTCTTACAAAGGCTTGTGTGGAAATGGAACAGAAAGTGTTAATTAATTACTATCCCACATGCCCCGAACCCGACCTGACGCTAGGTGTCAGAA   2496
AgFNSIc   AAAGAGGCTCTTACAAAGGCTTGTGTGGAAATGGAACAGAAAGTGTTAATTAATTACTATCCCACATGCCCCGAACCCGACTTGACGCTAGGTGTCAGAA   2472
AgFNSId   AAAGAGGCTCTTACAAAGGCTTGTGTGGAAATGGAACAGAAAGTGTTAATTAATTACTATCCCACATGCCCCGAACCCGACTTGACGCTAGGTGTCAGAA   2472
                                                                                                       AFGB-R

AgFNSIa   GGCATACGGATCCAGGTACTATTACCATTCTGCTTCAGGACATGGTTGGTGGTTTACAGGCTACTAGGGATGGCGGCAAAACTTGGATTACTGTTCAGCC   2598
AgFNSIb   GGCATACGGATCCAGGTACTATTACCATTCTGCTTCAGGACATGGTTGGTGGTTTACAGGCTACTAGGGATGGCGGCAAAACTTGGATTACTGTTCAGCC   2596
AgFNSIc   GGCATACGGATCCAGGTACTATTACCATTCTGCTTCAGGACATGGTTGGTGGTTTACAGGCTACTAGGGATGGCGGCAAAACTTGGATTACTGTTCAGCC   2572
AgFNSId   GGCATACGGATCCAGGTACTATTACCATTCTGCTTCAGGACATGGTTGGTGGTTTACAGGCTACTAGGGATGGCGGCAAAACTTGGATTACTGTTCAGCC   2572

                                                                             AFGA-R
AgFNSIa   TGTGGAGGGGAGCTTTTGTTGTCAATTTGGGTGATCATGGTCATGTAAGTTTGCTCTCTCCCTCTCTCTCTACCAATTTTGCTTAGTCTCTTGGTAAATGA   2698
AgFNSIb   TGTGGAGGGGAGCTTTTGTTGTCAATTTGGGTGATCATGGTCATGTAAGTTTGCTCTCTCCCTCTCTCTCTACCAATTTTGCTTAGTCTCTTGGTAAATGA   2696
AgFNSIc   TGTGGAGGGGAGCTTTTGTTGTCAATTTGGGTGATCATGGTCATGTAAGTTTGCTCTCTCCTTTTCTCTCTACCAATTTTGCTTAGTCTCTTGGTAAATGA   2672
AgFNSId   TGTGGAGGGGAGCTTTTGTTGTCAATTTGGGTGATCATGGTCATGTAAGTTTGCTCTCTCCTTTTCTCTCTACCAATTTTGCTTAGTCTCTTGGTAAATGA   2672

AgFNSIa   GGTGTATGTGGTATGTATAAAGTACTCCTATCTGCTGTAATTTATACTTAATCGTTTTAGAATGATTGATTTTGAATGAGCATATAGTTTAATTTAGGAA   2798
AgFNSIb   GGTGTATGTGGTATGTATAAAGTACTCCTATCTGCTGTAATTTATACTTAATCGTTTTAGAATGATTGATTTTGAATGAGCATATAGTTTAATTTAGGAA   2796
AgFNSIc   GGTGTATGTGGTATGTATAAAGTACTCCTATCTGCTGTAATTTATGCTTAATCGTTTTAGAATGATTGATTTTGAATGAGCATATAGTTTAATTTAGGAA   2772
AgFNSId   GGTGTATGTGGTATGTATAAAGTACTCCTATCTGCTGTAATTTATGCTTAATCGTTTTAGAATGATTGATTTTGAATGAGCATATAGTTTAATTTAGGAA   2772

AgFNSIa   TTTAGCTGACAACTTTGTGCTTTTATGTGTAGTATTTGAGCAATGGAAGGTTCAGGAATGCTGACCATCAAGCAGTAGTGAATTCAACTTCCACCAGATT   2898
AgFNSIb   TTTAGCTGACAACTTTGTGCTTTTATGTGTAGTATTTGAGCAATGGAAGGTTCAGGAATGCTGACCATCAAGCAGTAGTGAATTCAACTTCCACCAGATT   2896
AgFNSIc   TTTAGCTGACAACTTTGTGCTTTTATGTGTAGTATTTGAGCAATGGAAGGTTCAGGAATGCTGACCATCAAGCAGTAGTGAATTCAACTTCCACCAGATT   2872
AgFNSId   TTTAGCTGACAACTTTGTGCTTTTATGTGTAGTATTTGAGCAATGGAAGGTTCAGGAATGCTGACCATCAAGCAGTAGTGAATTCAACTTCCACCAGATT   2872

AgFNSIa   GTCAATTGCAACTTTCCAGAACCCGGCTCAGAATGCGATAGTATATCCGTTAAAGATCAGGGAGGGAGAGAAGGCAATTCTGGATGAGGCCATCACCTAC   2998
AgFNSIb   GTCAATTGCAACTTTCCAGAACCCGGCTCAGAATGCGATAGTATATCCGTTAAAGATCAGGGAGGGAGAGAAGGCAATTCTGGATGAGGCCATCACCTAC   2996
AgFNSIc   GTCAATTGCAACTTTCCAGAACCCGGCTCAGAATGCGATAGTATATCCGTTAAAGATCAGGGAGGGAGAGAAGGCAATTCTGGATGAGGCCATCACCTAC   2972
AgFNSId   GTCAATTGCAACTTTCCAGAACCCGGCTCAGAATGCGATAGTATATCCGTTAAAGATCAGGGAGGGAGAGAAGGCAATTCTGGATGAGGCCATCACCTAC   2972

AgFNSIa   GCTGAAATGTATAAGAAAAACATGACTAAACATATTGCGGTGGCTACCCAGAAGAAATTGGCCAAGGAGAAAAGGTTGCAAGATGAGAAGGCCAAGATGA   3098
AgFNSIb   GCTGAAATGTATAAGAAAAACATGACTAAACATATTGCGGTGGCTACCCAGAAGAAATTGGCCAAGGAGAAAAGGTTGCAAGATGAGAAGGCCAAGATGA   3096
AgFNSIc   GCTGAAATGTATAAGAAAAACATGACTAAACATATTGCGGTGGCTACCCAGAAGAAATTGGCCAAGGAGAAAAGGTTGCAAGATGAGAAGGCCAAGATGA   3072
AgFNSId   GCTGAAATGTATAAGAAAAACATGACTAAACATATTGCGGTGGCTACCCAGAAGAAATTGGCCAAGGAGAAAAGGTTGCAAGATGAGAAGGCCAAGATGA   3072

AgFNSIa   AGATATG   3105
AgFNSIb   AGATATG   3103
AgFNSIc   AGATATG   3079
AgFNSId   AGATATG   3079
```

Figure S1 Alignment of the alleles *AgFNSIa*, *AgFNSIb*, *AgFNSIc* and *AgFNSId*. Red and blue shadows indicate SNPs and InDels; Red box indicates start condons, and black boxes indicate introns; Underlines indicate primers of AFGA/AFGB, AFPA1/AFPB1 and AFPA2/AFPB2 markers.
