# Supplementary material for: Expression Analysis, Functional Marker Development and Verification of AgFNSI in Celery
Source: Sci Rep. 2020 Jan 17;10:531. doi: 10.1038/s41598-019-57054-x (PMC6969063; doi:10.1038/s41598-019-57054-x)
Supplement: Supplementary file 2 — Table S1. [file 41598_2019_57054_MOESM2_ESM.pdf]

Expression Analysis, Functional Marker Development and Verification of *AgFNSI* in Celery

Jun Yan, Li Yu, Lizhong He, Shuang Xu, Yanhui Wan, Hong Wang, Ying Wang, Weimin Zhu

Table S1 Predication analysis of the elements within the promoter of *AgFNSI*

Name of box	Sequence	Position (bp)	Function
A-box	CCGTCC	-803,-852	cis-acting regulatory element
ARE	AAACCA	-688	cis-acting regulatory element essential for the anaerobic induction
AT~TATA-box	TATATA	-149,-568,-388,-570	
Box 4	ATTAAT	-393,-699,-558,-1113	part of a conserved DNA module involved in light responsiveness
CAAT-box	CAAT	-89,-95,-216,-296,-489,-533,-539,-779,-789,-813,-1007,-1009,	
	CCAAT	-197,-778,-1006	common cis-acting element in promoter and enhancer regions
	CAAAT	-251,-451,-525,-900,-972,-1194,-1205, -1429	common cis-acting element in promoter and enhancer regions
CCGTCC motif	CCGTCC	-803,-852	
CGTCA motif	CGTCA	-102	cis-acting regulatory element involved in the MeJA-responsiveness
ERE	ATTTTAAA	-231	
GATA-motif	AAGGATAAGG	-137	part of a light responsive element
	GATAGGA	-912	part of a light responsive element
GC-motif	CCCCCG	-617	enhancer-like element involved in anoxic specific inducibility
GT1-motif	GGTTAA	-774	light responsive element
T-box	TGATAATGT	-109	part of a light responsive element
MBS	CAACTG	-1311	MYB binding site involved in drought-inducibility
MYB	TAACCA	-775	
	CAACCA	-1342	
MYB-like sequence	TAACCA	-775	
MYC	CATTTG	-971,-1194,-1204	
	CAATTG	-1007	
Myb	CAACTG	-1311	
STRE	AGGGG	-309,-436	
TATA-box	ATATAA	-47,-467,-571,-161,-863,-1106	core promoter element around -30 of transcription start
	ATATAT	-389,-569,-150,-176,-589,-1350	core promoter element around -30 of transcription start
	TATATA	-388,-568,-149,-570	core promoter element around -30 of transcription start

TATA	-390,-48,-463,-468,-522,-126,-151,-572,-162,-177,-238,-285,-348,-582,-590,-822,-829,-864,-1090,-1101,-1107,-1285,-1351,-1455	core promoter element around -30 of transcription start
