# Supplementary material for: Expression Analysis, Functional Marker Development and Verification of AgFNSI in Celery
Source: Sci Rep. 2020 Jan 17;10:531. doi: 10.1038/s41598-019-57054-x (PMC6969063; doi:10.1038/s41598-019-57054-x)
Supplement: Supplementary file 3 — Table S2. [file 41598_2019_57054_MOESM3_ESM.pdf]

Expression Analysis, Functional Marker Development and Verification of *AgFNSI* in Celery

Jun Yan, Li Yu, Lizhong He, Shuang Xu, Yanhui Wan, Hong Wang, Ying Wang, Weimin Zhu

Table S2 SNP and Haplotype in promoter of *AgFNSI*

Haplotype Type		AgFNSIa	AgFNSIb	AgFNSIc	AgFNSId
Material number		4	2	5	2
Frequency of Haplotype (%)		30.76%	15.38%	38.46%	15.38%
SNP (bp) in promoter region	-11	T	T	T	C
	-86	A	A	C	C
	-98	G	G	A	A
	-102	T	T	G	G
	-127—-128	AT	AT	-	-
	-159	T	T	C	C
	-251	A	T	T	T
	-310	G	G	A	A
	-321	T	T	C	C
	-328	G	G	A	A
	-344	C	T	T	T
	-385	G	G	T	T
	-419	G	A	G	G
	-430	T	C	C	C
	-437—-439	CCC	CCC	-	-
	-535	A	A	-	-
	-653	C	C	T	T
	-689	A	A	T	T
	-761	T	T	A	A
	-768	T	T	C	C
	-769	C	C	T	T
	-953	T	T	C	C
	-955	C	C	A	A
	-1031	A	A	A	G
	-1039	G	G	A	A
	-1046	G	G	A	A
	-1059	C	T	T	T
	-1069	T	A	A	A
	-1091	C	C	T	T
	-1103—-1104	AT	AT	-	-
	-1116	T	T	-	-
	-1138	C	C	T	T
	-1141	C	C	T	T
	-1155—-1165	TTTTTCTTAAA	TTTTTCTTAAA	-	-
	-1201	G	G	A	A
	-1234—-1236	CGG	CGG	-	-
	-1250	T	T	-	-
SNP (bp) in genome region	-1501	T	T	T	A
	-1502	A	A	T	T
	-1522	T	T	C	C

	-1524	T	T	C	C
	-1604	A	A	a	G
	-1777	T	T	C	C
	-1819	A	A	A	G
	-1884	A	G	A	A
	-1887	A	T	A	A
	-1895	C	T	C	C
	-1912	T	T	C	C
	-1917	C	C	T	T
	-1918	T	T	A	A
	-1933	T	T	A	A
	-1974	T	T	C	C
	-2018—-2019	GG	-	-	-
	-2045—-2046	GA	GA	-	-
	-2107—-2018	-	-	AA	AA
	-2143	C	A	A	A
	-2170	C	C	T	T
	-2192	T	G	G	G
	-2259	G	G	C	C
	-2268	C	C	T	T
	-2282	A	A	G	G
	-2482	C	C	T	T
	-2661	C	C	T	T
	-2663	C	C	T	T
	-2746	A	A	G	G
