# Supplementary material for: Expression Analysis, Functional Marker Development and Verification of AgFNSI in Celery
Source: Sci Rep. 2020 Jan 17;10:531. doi: 10.1038/s41598-019-57054-x (PMC6969063; doi:10.1038/s41598-019-57054-x)
Supplement: Supplementary file 4 — Table S3. [file 41598_2019_57054_MOESM4_ESM.pdf]

Expression Analysis, Functional Marker Development and Verification of *AgFNSI* in Celery

Jun Yan, Li Yu, Lizhong He, Shuang Xu, Yanhui Wan, Hong Wang, Ying Wang, Weimin Zhu

Table S3 The names and origins of 112 celery accessions

Number	Accession ID	Name	Seed origin
1	PI601133	Ventura	U.S. National Plant Germplasm System
2	PI601148	Napolean	
3	PI542396	Matador	
4	PI545482	Gene's Gem 11-7	
5	PI587186	Comet	
6	PI662499	Delmar	
7	PI662473	Golden-Self Blanching	
8	PI662474	June Belle	
9	PI662490	Utah Early Green	
10	PI662491	Spartan 162	
11	PI662492	Tall Utah 52-70 H	
12	PI662493	Tall Fordhook 38147	
13	PI662494	Erfurt	
14	PI662495	Golden Plume 4162	
15	PI662496	Tall Fordhook	
16	PI662500	Emerald	
17	PI662501	Florimart	
18	PI662502	Giant Pascal	
19	PI662503	Florida Green Pascal	
20	PI662504	Florida 2-13(20-70)	
21	PI662505	Penn H-46	
22	PI662506	Florida 683	
23	PI662507	Emerson Pascal	
24	PI662508	S48-54-1	
25	PI662509	Tall Green Light	
26	PI662511	Beacon	
27	PI662512	Glademaster 136	
28	PI662463	Cornell 19	
29	PI662465	378 Summer Pascal	
30	PI662467	Bishop	
31	PI601659	Hercules	
32	PI662497	Advantage	
33	PI662514	Golden Spartan	
34	PI662498	Companion	
35	PI174051	7765	
36	PI175591	G 4279	
37	PI175593	9716	
38	PI662470	Xia Qin	
39	AG001	Huangxin Qin	Chinese Local Varieties
40	AG002	Zhangqiu Bao Qin	
41	AG003	Bolicui Shiqin	
42	AG004	Xingjiang Small Mao Qin	
43	AG005	Juhua Big leaf hollow Qin	
44	AG006	Huxin Miaoqin	

45	AG007	Yellow seedling Qin	
46	AG008	Tiegan Qincai	
47	AG009	Qijia Qincai	
48	AG010	Jingjia Shiganqincai	
49	AG011	Qichun Qingqincai	
50	AG012	Nongli Qingqin	
51	AG013	Chongxin Qincai	
52	AG014	Shixin Qincai	
53	AG015	Dianji Qincai	
54	AG016	Chunfeng Qincai	
55	AG017	Qingmiao Shixinqin	
56	AG018	Guiyang Whiteqin	
57	AG019	Guangzhou Whiteqin	
58	AG020	Sichuan Whiteqin	
59	AG021	Shanxi purple Qin	
60	AG022	Shenjie Whiteqin	
61	AG023	Shangnong yellow leaf Qin	Shanghai Academy of Agricultural Sciences
62	AG024	ShenQin No.1	
63	AG025	ShenQin No.2	
64	AG026	Jinqin No.36	Tianjin Kexing Vegetable Research Institute
65	AG027	Jinqin No.13	
66	AG028	Jinqi No.1	Tianjin Hongcheng Celery Research Institute
67	AG029	Little angel	
68	AG030	Sijiqingxiang little Qin	
69	AG031	Jinnanshiqin No. 1	
70	AG032	Jinnanshiqin No. 2	
71	AG033	Jinnanshiqin No. 3	
72	AG034	White petiole Qin	
73	AG035	Hangyu No.1	
74	AG036	Hangyu No.2	
75	AG037	Hangyu No.3	
76	AG038	Hongcheng hollow Qin	
77	AG039	Baimiao Qincai	
78	AG040	Tango	Netherlands Bejo Seed Co., Ltd.
79	AG041	Samba	
80	AG042	Yuhuang	Rijk Zwaan (China) Seed Co., Ltd.
81	AG043	Kelvin	
82	AG044	Shangnong Yuqin	Shandong Vegetable Seed Congress and Beijing Seed Congress
83	AG045	Yuxiang No.1	
84	AG046	Xiyu No.1	
85	AG047	LongQin NO.1	
86	AG048	King yellow	
87	AG049	Cornell 619	
88	AG050	Small celery	
89	AG051	Huangfei	
90	AG052	Pioneer	

91	AG053	Prince
92	AG054	Niederlan
93	AG055	Queen
94	AG056	Zeus
95	AG057	Huangnen celery
96	AG058	Ferrari
97	AG059	Delit
98	AG060	PRINCESS
99	AG061	Yameixiangqin
100	AG062	Emperor
101	AG063	Sushengxiaoxiangyu
102	AG064	Rome P9
103	AG065	Rafi
104	AG066	Holy Emperor
105	AG067	Cuiyu
106	AG068	Crown
107	AG069	Caesar
108	AG070	Hanchun celery
109	AG071	Noble celery
110	AG072	Blush
111	AG073	Betty
112	AG074	California King

The seeds of number 39-112 accessions were collected and conserved in Celery Germplasm Resource Bank of Shanghai academy of Agricultural Sciences. Number 39-60 accessions were Chinese Local Varieties, which were collected from local farmers at different countryside in China; number 82-112 accessions were purchased by our research team at Shandong Vegetable Seed Congress and Beijing Seed Congress in China.

The accession ID begin with I was the same as it in U.S. National Plant Germplasm System, and the accession ID from AG001 to AG074 was numbered by our research team.
